# Data-driven design of targeted gene panels for estimating immunotherapy biomarkers

**DOI:** 10.1038/s42003-022-03098-1

**Published:** 2022-02-23

**Authors:** Jacob R. Bradley, Timothy I. Cannings

**Affiliations:** School of Mathematics, University of Edinburgh, Edinburgh, UK

**Keywords:** Cancer genomics, Tumour biomarkers, Statistical methods

## Abstract

Tumour mutation burden and other exome-wide biomarkers are used to determine which patients will benefit from immunotherapy. However, the cost of whole exome sequencing limits the widespread use of such biomarkers. Here, we introduce a data-driven framework for the design of targeted gene panels for estimating a broad class of biomarkers including tumour mutation burden and tumour indel burden. Our first goal is to develop a generative model for the profile of mutation across the exome, which allows for gene- and variant type-dependent mutation rates. Based on this model, we then propose a procedure for constructing biomarker estimators. Our approach allows the practitioner to select a targeted gene panel of prespecified size and construct an estimator that only depends on the selected genes. Alternatively, our method may be applied to make predictions based on an existing gene panel, or to augment a gene panel to a given size. We demonstrate the excellent performance of our proposal using data from three non small-cell lung cancer studies, as well as data from six other cancer types.

## Abstract (web summary)

A data-driven procedure to estimate immunotherapy biomarkers is shown to perform well on data from three Non-Small Cell Lung Cancer studies as well as data from six other cancer types. The method is based on a generative model of how mutations arise in the tumour exome.

## Introduction

It has been understood for a long time that cancer, a disease occurring in many distinct tissues of the body and giving rise to a wide range of presentations, is initiated and driven by the accumulation of mutations in a subset of a person’s cells^1^. Since the discovery of immune checkpoint blockade (ICB)^2–4^, there has been an explosion of interest in cancer therapies targeting immune response and ICB therapy is now widely used in clinical practice^5^. ICB therapy works by targeting natural mechanisms (or *checkpoints*) that disengage the immune system, for example, the proteins cytotoxic T-lymphocyte-associated protein 4 (CTLA-4) and programmed death ligand 1 (PD-L1)^6^. Inhibition of these checkpoints can promote a more aggressive anti-tumour immune response^7^, and in some patients, this leads to long-term remission^8^. However, ICB therapy is not always effective^9^ and may have adverse side-effects, so determining which patients will benefit in advance of treatment is vital.

Exome-wide prognostic biomarkers for immunotherapy are now well-established—in particular, tumour mutation burden (TMB) is used to predict response to immunotherapy^10,11^. TMB is defined as the total number of non-synonymous mutations occurring throughout the tumour exome, and can be thought of as a proxy for how easily a tumour cell can be recognised as foreign by immune cells^12^. However, the cost of measuring TMB using whole exome sequencing (WES)^13^ currently prohibits its widespread use as standard-of-care. Sequencing costs, both financial and in terms of the time taken for results to be returned, are especially problematic in situations where high-depth sequencing is required, such as when utilising blood-based circulating tumour DNA (ctDNA) from liquid biopsy samples^14^. The same issues are encountered when measuring more recently proposed biomarkers such as tumour indel burden (TIB)^15,16^, which counts the number of frameshift insertion and deletion mutations. There is, therefore, demand for cost-effective approaches to estimate these biomarkers^17,18^.

In this paper, we propose a data-driven method for biomarker estimation, based on a generative model of how mutations arise in the tumour exome. More precisely, we model mutation counts as independent Poisson variables, where the mean number of mutations depends on the gene of origin and variant type, as well as the background mutation rate (BMR) of the tumour. Due to the ultrahigh-dimensional nature of sequencing data and the fact that in many genes mutations arise purely according to the BMR, we use a regularisation penalty when estimating the parameters of the model. In addition, this identifies a subset of genes that are mutated above or below the background rate. Our model facilitates the construction of an estimator of TMB, based on a weighted linear combination of the number of mutations in each gene. The vector of weights is chosen to be sparse (i.e. have many entries equal to zero), so that our estimator of TMB may be calculated using only the mutation counts of a subset of genes. In particular, this allows for accurate estimation of TMB from a targeted gene panel, where the panel size (and therefore the cost) may be determined by the user. We also provide an R package ICBioMark^19^ which implements the methodology and reproduces the experimental results in the paper.

We demonstrate the excellent practical performance of our framework using a non-small cell lung cancer (NSCLC) dataset^20^, and include a comparison with existing state-of-the-art approaches for estimating TMB. We further validate these results by testing the performance on data from two more NSCLC studies^21,22^. Moreover, since our model allows variant type-dependent mutation rates, it can be adapted easily to predict other biomarkers, such as TIB. Our method may also be used in combination with an existing targeted gene panel. In particular, we can estimate a biomarker directly from the panel, or first augment the panel and then construct an estimator. Finally, in order to further investigate the utility of our proposal across a range of mutation profiles, we use it to select targeted gene panels and estimate TMB in six other cancer types.

Due to its emergence as a biomarker for immunotherapy in recent years, a variety of groups have considered methods for estimating TMB. A simple and common way to estimate TMB is via the proportion of mutated codons in a targeted region. Budczies et al.^23^. investigate how the accuracy of predictions made in this way are affected by the size of the targeted region, where mutations are assumed to occur at a uniform rate throughout the genome. More recently Yao et al.^24^ modelled mutations as following a negative binomial distribution while allowing for gene-dependent rates, which are inferred by comparing non-synonymous and synonymous mutation counts. In contrast, our method does not require data including synonymous mutations. Where they are included, we do not assume that synonymous mutations occur at a uniform rate throughout the genome, giving us the flexibility to account for location-specific effects on synonymous mutation rates such as chromatin configuration^25^ and transcription-dependent repair mechanisms^26^. Linear regression models have been used for both panel selection^27^ and for biomarker prediction^28^. A review of some of the issues arising when dealing with targeted panel-based predictions of TMB biomarkers is given by Wu et al.^29^. Finally, we are unaware of any methods for estimating TIB from targeted gene panels.

## Results and discussion

In this section, we demonstrate in detail the practical performance of our proposal using the dataset from Campbell et al.^20^. Our main focus is the prediction of TMB, and we show that our method outperforms state-of-the-art approaches. We also analyse the suitability of our generative model, include a panel augmentation case study with the TST-170 gene panel, and consider the task of predicting the recently proposed biomarker TIB. Finally, in this section, we test our method’s generality and robustness by applying it to data from two further NSCLC datasets and then six further cancer types.

### Data and terminology

**Fig. 1:**
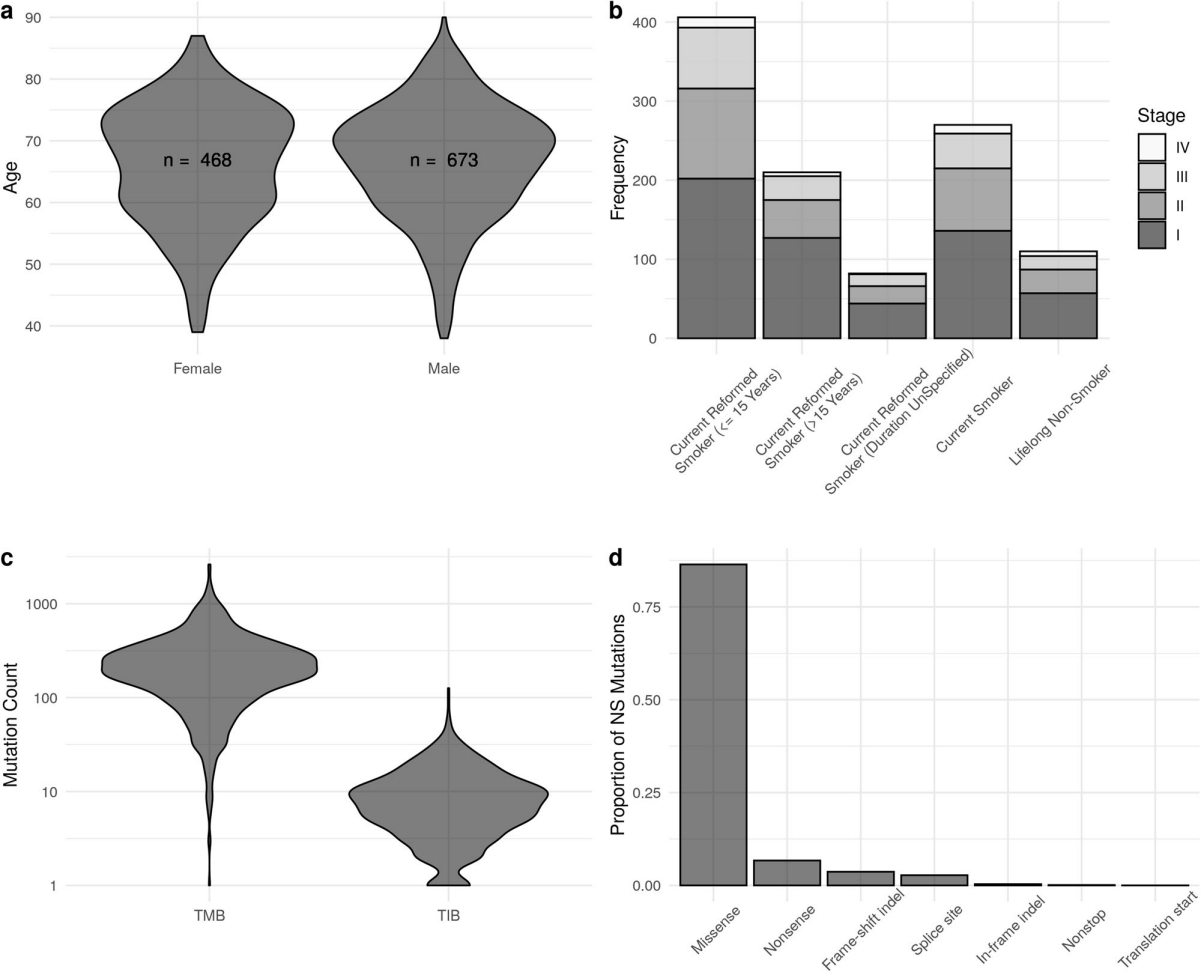
Population data for the clinical cohort in Campbell et al. NSCLC dataset^20^. **a** Violin plots of age for patients, stratified by sex. **b** Stacked bar chart of patients' smoking histories, shaded according to cancer stage diagnosis. **c** Violin plot of the distribution of TMB and TIB across training samples. **d** The relative frequency of different non-synonymous mutation types.

Our methodology can be applied to any annotated mutation dataset obtained by WES. To demonstrate our proposal we make use of the NSCLC dataset produced by Campbell et al.^20^, which contains data from 1144 patient-derived tumours. For each sample in this dataset, we have the genomic locations and variant types of all mutations identified. At the time of the study, the patients had a variety of prognoses and smoking histories, were aged between 39 and 90, 41% were female and 59% were male; see Fig. 1a, b. In Fig. 1c we see that mutations counts are distributed over a very wide range, as is the case in many cancer types^30^. For simplicity, we only consider seven non-synonymous variant types: missense mutations (which are the most abundant), nonsense mutations, frameshift insertions/deletions, splice site mutations, in-frame insertions/deletions, nonstop mutations and translation start site mutations. We present the frequencies of these mutation types in Fig. 1d. Frameshift insertion/deletion (also known as indel) mutations are of particular interest when predicting TIB, but contribute only a small proportion (<4%) of non-synonymous mutations.

It is useful at this point to introduce the notation used throughout the paper. The set *G* denotes the collection of genes that make up the exome. For a gene *g* ∈ *G*, let *ℓ*_*g*_ be the length of *g* in nucleotide bases, defined by maximum coding sequence as collected from the *Ensembl* database^31^. A gene panel is a subset *P* ⊆ *G*, and we write *ℓ*_*P*_ ≔ ∑_*g*∈*P*_*ℓ*_*g*_ for its total length. We let *S* denote the set of variant types in our data (e.g. in the dataset mentioned above, *S* contains the seven possible non-synonymous variants). Now, for *i* = 0, 1, …, *n*, let *M*_*igs*_ denote the count of mutations in gene *g* ∈ *G* of type *s* ∈ *S* in the *i*th sample. Here the index *i* = 0 is used to refer to an unseen test sample for which we would like to make a prediction, while the indices *i* = 1, …, *n* enumerate the samples in our training dataset. In order to define the exome-wide biomarker of particular interest, we specify a subset of mutation types $\bar{S}\subseteq S$, and let

1 $${T}_{i\bar{S}}:= \mathop{\sum}\limits_{g\in G}\mathop{\sum}\limits_{s\in \bar{S}}{M}_{igs},$$

for *i* = 0, …, *n*. For example, including all non-synonymous mutation types in $\bar{S}$ specifies ${T}_{i\bar{S}}$ as the TMB of sample *i*, whereas letting $\bar{S}$ contain only indel mutations gives TIB.

Our main goal is to predict ${T}_{0\bar{S}}$ based on {*M*_0*gs*_: *g* ∈ *P*, *s* ∈ *S*}, where the panel *P* ⊆ *G* has length *ℓ*_*P*_ satisfying some upper bound. When it is clear from context that we are referring to the test sample and a specific choice of biomarker (i.e. $\bar{S}$ is fixed), we will simply write *T* in place of ${T}_{0\bar{S}}$.

Since we are only looking to produce estimators for TMB and TIB, we group mutations into two categories—indel mutations and all other non-synonymous mutations—so that ∣*S*∣ = 2. This simplifies the presentation of our results and reduces the computational cost of fitting the generative model. In order to assess the performance of each of the methods in this section, we randomly split the dataset into training, validation and test sets, which contain *n*_train_ = *n* = 800, *n*_val_ = 171 and *n*_test_ = 173 samples, respectively. Mutations are observed in ∣*G*∣ = 17,358 genes. Our training set comprises samples with an average TMB of 252 and TIB of 9.25.

### Generative model fit

**Fig. 2:**
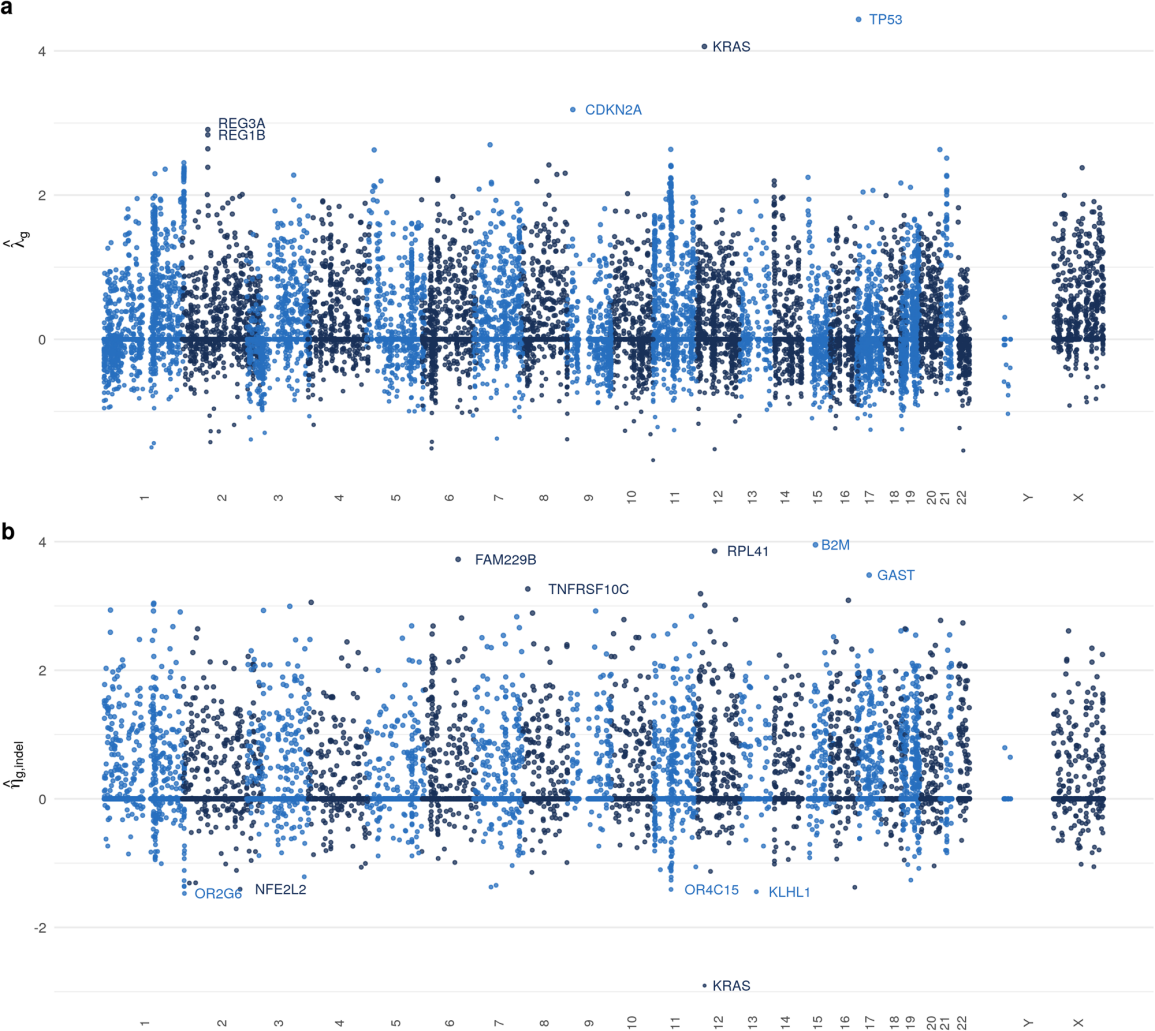
Manhattan plots of generative model parameters. **a** Manhattan plot of fitted parameters ${\hat{\lambda }}_{g}$ and their associated genes' chromosomal locations. The genes with the five largest positive parameter estimates are labelled. **b** Manhattan plot of fitted parameters ${\hat{\eta }}_{g,{{{{\rm{indel}}}}}}$ and their associated genes' chromosomal locations. The five largest positive and negative genes are labelled.

The first step in our analysis is to fit our generative model using only the training dataset. In particular, we obtain estimates of the model parameters using equation (4), where the tuning parameter *κ*_1_ is determined using tenfold cross-validation as described in the Methodology section. Diagnostics and model validation statistics are presented in Supplementary Fig. 1 and Supplementary Table 1. The best choice of *κ*_1_ produces estimates of *λ* and *η* with 44.4 and 77.8% sparsity respectively, i.e. that proportion of their components are estimated to be exactly zero. We plot $\hat{\lambda }$ and $\hat{\eta }$ for this value of *κ*_1_ in Fig. 2a, b. Genes with ${\hat{\lambda }}_{g}=0$ are interpreted to be mutating according to the background mutation rate, and genes with ${\hat{\eta }}_{{{{{\rm{g,indel}}}}}}=0$ are interpreted as having no specific selection pressure for or against indel mutations. In Fig. 2a, b we highlight genes with large (in absolute value) parameter estimates, some of which have known biological relevance in oncology; see our Conclusion for further discussion. Finally, note that the average fitted value of *μ*_*i*_ among current smokers is 5.40 (with a standard deviation of 0.76), amongst reformed smokers is 5.26 (0.84), and among lifelong non-smokers is 4.04 (1.12). This suggests that smokers may have higher BMRs, as would be expected.

### Predicting tumour mutation burden

We now demonstrate the practical performance of our procedure for estimating TMB. First, it is shown that our method can indeed select gene panels of the size specified by the practitioner and those good predictions can be made even with small panel sizes (i.e. ≤1 Mb). We then compare the performance of our proposal with state-of-the-art estimation procedures based on a number of widely used gene panels.

In order to evaluate the predictive performance of an estimator we calculate the *R*^2^ score on the validation data as follows: given predictions of TMB, ${\hat{t}}_{1},\ldots ,{\hat{t}}_{{n}_{val}}$, for the observations in the validation set with true TMB values ${t}_{1},\ldots ,{t}_{{n}_{val}}$. Let $\bar{t}:= \frac{1}{{n}_{val}}\mathop{\sum }\nolimits_{i = 1}^{{n}_{val}}{t}_{i}$, and define

$${R}^{2}:= 1-\frac{\mathop{\sum }\nolimits_{i = 1}^{{n}_{val}}{({t}_{i}-{\hat{t}}_{i})}^{2}}{\mathop{\sum }\nolimits_{i = 1}^{{n}_{val}}{({t}_{i}-\bar{t})}^{2}}.$$

Other works have aimed to classify tumours into two groups (high TMB, low TMB); see, for example, Buttner et al.^32^ and Wu et al.^29^. Here we also report the estimated area under the precision-recall curve (AUPRC) for a classifier based on our estimator. We define the classifier as follows: first, in line with major clinical studies^33,34^ the true class membership of a tumour is defined according to whether it has t^*^ ≔ 300 or more exome mutations (~10 Mut/Mb). In the validation set, this gives 47(27.5%) tumours with high TMB and 124(72.5%) with low TMB. Now, for a cutoff *t* ≥ 0, we can define a classifier by assigning a tumour to the high TMB class if its estimated TMB value is greater than or equal to *t*. For such a classifier, we have precision and recall (estimated over the validation set) given by

$$p(t):= \frac{\mathop{\sum }\nolimits_{i = 1}^{{n}_{val}}{{\mathbb{1}}}_{\{{\hat{t}}_{i}\ge t,{t}_{i}\ge {t}^{* }\}}}{\mathop{\sum }\nolimits_{i = 1}^{{n}_{val}}{{\mathbb{1}}}_{\{{\hat{t}}_{i}\ge t\}}}\quad \,{{{{\rm{and}}}}}\,\quad r(t):= \frac{\mathop{\sum }\nolimits_{i = 1}^{{n}_{val}}{{\mathbb{1}}}_{\{{\hat{t}}_{i}\ge t,{t}_{i}\ge {t}^{* }\}}}{\mathop{\sum }\nolimits_{i = 1}^{{n}_{val}}{{\mathbb{1}}}_{\{{t}_{i}\ge {t}^{* }\}}},$$

respectively. The precision-recall curve then is {(*r*(*t*), *p*(*t*)): *t* ∈ [0, *∞*)}. Note that a perfect classifier achieves a AUPRC of 1, whereas a random guess, in this case, would have an average AUPRC of 0.275 (the prevalence of the high TMB class).

Now recall that TMB is given by equation (1) with $\bar{S}$ being the set of all non-synonymous mutation types. Thus to estimate TMB we apply our proposed estimator with $\bar{S}=S$, where the model parameters are estimated as described in our generative model section. In Supplementary Fig. 2, we present the *R*^2^ and AUPRC for the first-fit and refitted estimators (see (6) and (8)) as the selected panel size varies from 0Mb to 2 Mb in length. We see that we obtain a more accurate prediction of TMB, both in terms of regression and classification, as the panel size increases, and that good estimation is possible even with very small panels (as low as 0.2 Mb). As expected, the refitted estimator slightly outperforms the first-fit estimator. We show good robustness of these results to permutations of the training set in Supplementary Fig. 3.

We now compare our method with state-of-the-art estimators applied to commonly used gene panels, as well as a panel selected by the proposal of Lyu et al.^27^. The three next-generation sequencing panels that we consider are chosen for their relevance to TMB. These are TST-170^35^, Foundation One^36^ and MSK-IMPACT^37^. Further, the panel selected by the approach in Lyu et al.^27^ consists of the genes that are mutated more than 10% of the time, that are less than 0.015 Mb in length and for which the presence of a mutation in the gene is significantly associated with higher TMB values. For each panel *P* ⊆ *G*, we use four different methods to predict TMB:

(i) Our refitted estimator applied to the panel *P*: we estimate TMB using $T({\hat{w}}_{P})$, where ${\hat{w}}_{P}\in {{{{{{\rm{argmin}}}}}}}_{w\in {W}_{P}}\{f(w)\}$, and *W*_*P*_ is defined in (7).
(ii) Estimation and classification of tumour mutation burden (ecTMB): the procedure proposed by Yao et al.^24^.
(iii) A count estimator: TMB is estimated by $\frac{{\ell }_{G}}{{\ell }_{P}}{\sum }_{g\in P}{\sum }_{s\in \bar{S}}{M}_{0gs}$, i.e. rescaling the mutation burden in the genes of *P*.
(iv) A linear model: we estimate TMB via ordinary least-squares linear regression of TMB against $\big\{{\sum }_{s\in S}{M}_{0gs}:g\in P\big\}$.

The latter three comprise existing methods for estimating TMB available to practitioners. The second (ecTMB), which is based on a negative binomial model, is state-of-the-art. The third is a standard practical procedure for the estimation of TMB from targeted gene panels. The fourth is the approach proposed by Lyu et al.^27^. The refitted estimator applied to panel *P* is also included here, in order to demonstrate the utility of our approach even with a prespecified panel.

**Fig. 3:**
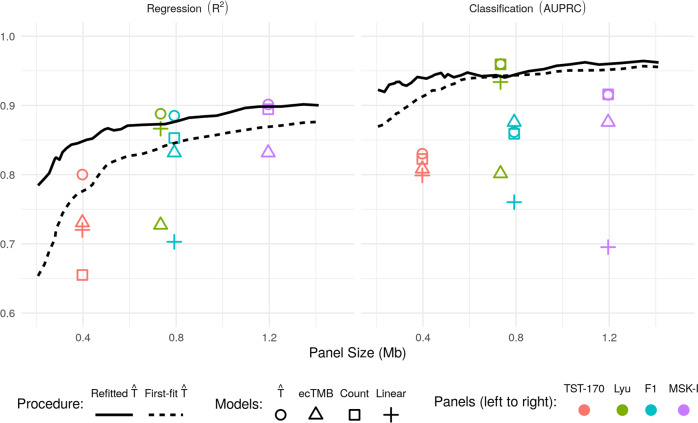
Comparison with existing estimators. The performance of our TMB estimator in comparison to existing approaches. Left: *R*^2^, Right: AUPRC.

We present the results of these comparisons in Fig. 3. First, for each of the four panels considered here, we see that our refitted estimator applied to the panel outperforms all existing approaches in terms of regression performance and that for smaller panels we are able to improve regression accuracy even further by selecting a panel (perhaps even of smaller size) based on the training data. For instance, in comparison to predictions based on the TST-170 panel, our procedure can achieve higher *R*^2^ with a selected panel of half the size (with 0.2 Mb we obtain an *R*^2^ of 0.78). The best available existing method based on the TST-170 panel, in this case, the linear estimator, has an *R*^2^ of 0.74. Moreover, data-driven selection of panels considerably increases the classification performance for the whole range of panel sizes considered. In particular, even for the smallest panel size shown in Fig. 3 (~0.2 Mb), the classification performance of our method outperforms the best existing methodology applied to the MSK-IMPACT panel, despite being almost a factor of six times smaller. The full proposal of Lyu et al.^27^, which involves applying the linear regression estimate to the panel selected as described above, also performs well here.

**Fig. 4:**
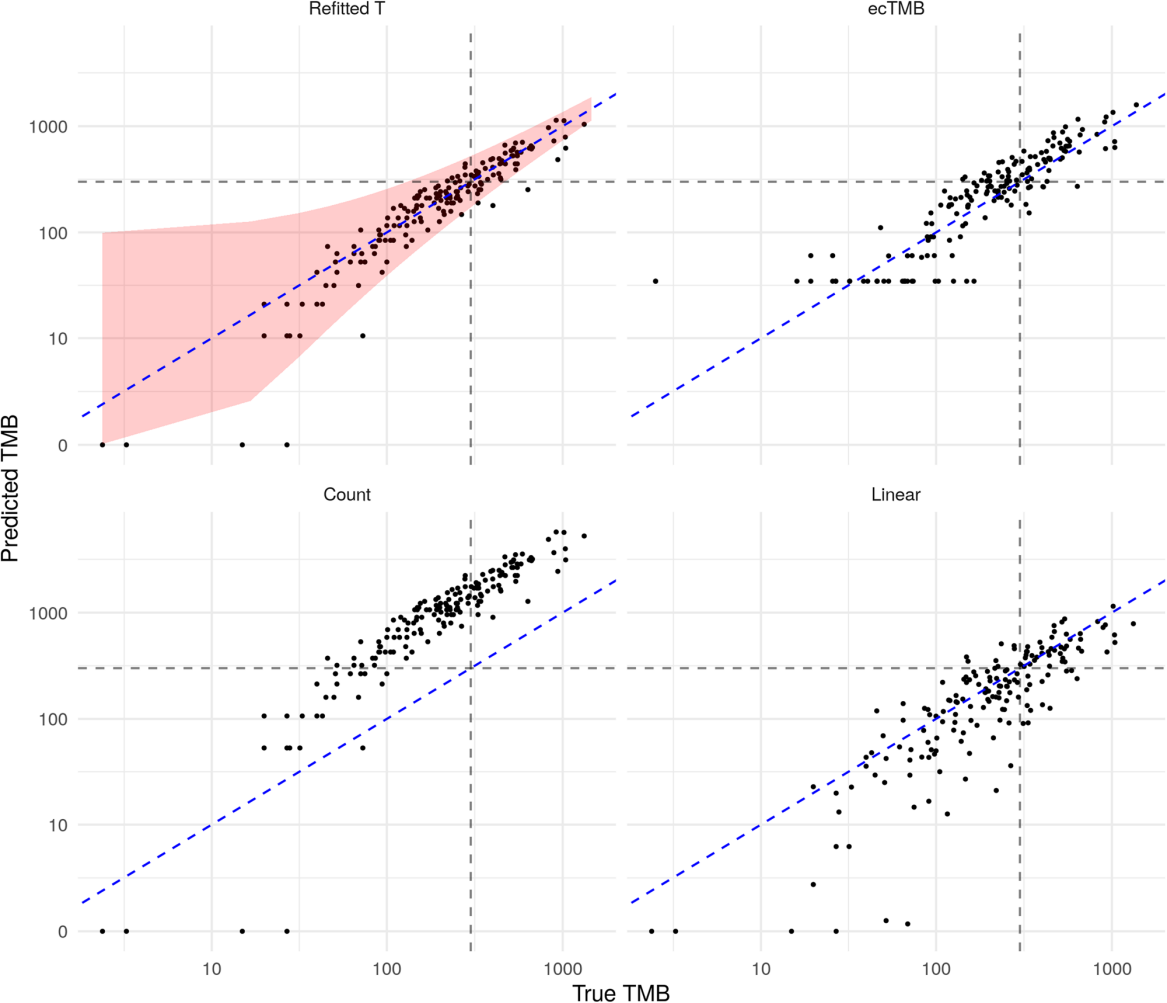
Prediction of TMB on the test dataset. Dashed blue (diagonal) line represents perfect prediction and the black dashed lines indicate true and predicted TMB values of 300.

Finally, in this subsection, we demonstrate the practical performance of our method using the test set, which until this point has been held out. Based on the validation results above, we take the panel of size 0.6 Mb selected by our procedure and use our refitted estimator on that panel to predict TMB for the 173 samples in the test set. For comparison, we also present predictions from ecTMB, the count-based estimator and the linear regression estimator applied to the same panel. In Fig. 4 we see that our procedure performs well; we obtain an *R*^2^ value (on the test data) of 0.85. The other methods have *R*^2^ values of 0.67 (ecTMB), −36 (count) and 0.64 (linear regression). The count-based estimator here gives predictions which are reasonably well correlated to the true values of TMB but are positively biased. This is because our selection procedure tends to favour genes with higher overall mutation rates and thus a count estimator based on the highly mutated genes will overestimate the total number of mutations. We also include a red shaded region comprising all points for which heuristic 90% prediction intervals (as described in our Practical considerations section) include the true TMB value. We find in this case that 93.6% of the observations in the test set fall within this region, giving valid empirical coverage.

### A panel-augmentation case study

We may wish to include genes from a given panel, but use our methodology to augment the panel to include additional genes with the goal of obtaining more accurate predictions of TMB (or other biomarkers). In this section we demonstrate how this can be done starting with the TST-170 panel (~0.4 Mb) and augmenting to 0.6 Mb in length, demonstrating impressive gains in predictive performance.

We apply the augmentation method described in the Panel augmentation methods section, with *P*_0_ taken to be the set of TST-170 genes and *Q*_0_ to be empty. The genes added to the panel are determined by the first-fit estimator in equation (9). To evaluate the performance, we then apply the refitted estimator on the test dataset, after selecting the augmented panel of size 0.6 Mb. For comparison, we apply our refitted estimator to the TST-170 panel directly. We also present the results obtained by the other estimators described above, both before and after the panel augmentation, in Table 1. We find that by augmenting the panel we improve predictive performance with our refitted $\hat{T}$ estimator, both in terms of regression and classification. The refitted estimator provides better estimates than any other model on the augmented panel by both metrics.

**Table 1:** Predictive performance of models on TST-170 (0.4 Mb) versus augmented TST-170 (0.6 Mb) panels on the test set.

Model	Regression (*R*^2^)		Classification (AUPRC)	
	TST-170	Aug. TST-170	TST-170	Aug. TST-170
Refitted $\hat{T}$	**0.58**	**0.84**	**0.84**	**0.94**
ecTMB	0.37	0.51	0.80	0.88
Count	0.18	0.18	0.83	**0.94**
Linear	0.47	0.74	0.78	0.89

Highest values for each column are in bold.

### Predicting tumour indel burden

In this section, we demonstrate how our method can be used to estimate TIB. This is more challenging than estimating TMB due to the low abundance of indel mutations relative to other variant types (see Fig. 1d), as well as issues involved in sequencing genomic loci of repetitive nucleotide constitution^38^. Indeed, in contrast to the previous section, we are not aware of any existing methods designed to estimate TIB from targeted gene panels. We, therefore, investigate the performance of our method across a much wider range (0–30 Mb) of panel sizes and find that we are able to accurately predict TIB with larger panels. Our results also demonstrate that accurate classification of TIB status is possible even with small gene panels.

We let *S*_indel_ be the set of all frameshift insertion and deletion mutations, and apply our method introduced in the methods section with $\bar{S}={S}_{{{{{\rm{indel}}}}}}$. As in the previous section, we assess regression and classification performance via *R*^2^ and AUPRC, respectively, where in this case tumours are separated into two classes: high TIB (ten or more indel mutations) and low TIB (otherwise). In the validation dataset, this gives 57(33.3%) tumours in the high TIB class.

**Fig. 5:**
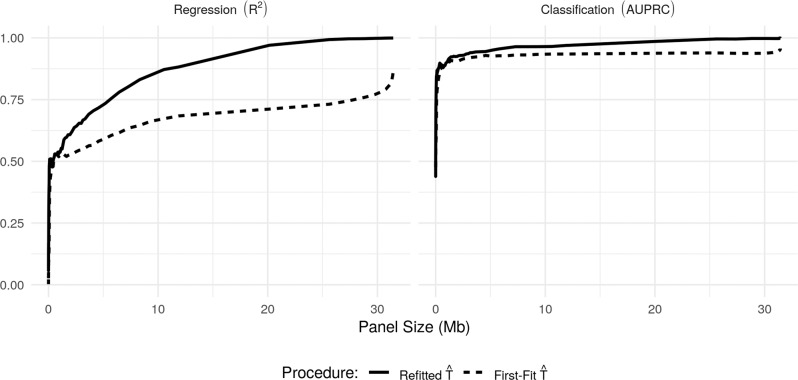
Estimating TIB on the validation dataset. Performance of our first-fit and refitted estimators of TIB as the selected panel size varies. Left: *R*^2^, Right: AUPRC.

The results are presented in Fig. 5. We comment first on the regression performance: as expected, we see that the *R*^2^ values for our first-fit and refitted estimators are much lower than what we achieved in estimating TMB. The refitted approach improves for larger panel sizes, while the first-fit estimator continues to perform relatively poorly. On the other hand, we see that the classification performance is impressive, with AUPRC values of above 0.8 for panels of less than 1 Mb in size.

We now assess the performance on the test set of our refitted estimator of TIB applied to a selected panel of size 0.6 Mb, and we compare with a count-based estimator and linear regression estimator. We do not compare with ecTMB here, since it is designed to estimate TMB as opposed to TIB. The count-based estimator, in this case, scales the total number of non-synonymous mutations across the panel by the ratio of the length of the panel to that of the entire exome, and also by the relative frequency of indel mutations versus all non-synonymous mutations in the training dataset:

$$\frac{{\ell }_{G}}{{\ell }_{P}}\frac{\mathop{\sum }\nolimits_{i = 1}^{n}{\sum }_{g\in G}{\sum }_{s\in {S}_{{{{{\rm{indel}}}}}}}{M}_{igs}}{\mathop{\sum }\nolimits_{i = 1}^{n}{\sum }_{g\in G}{\sum }_{s\in S}{M}_{igs}}\mathop{\sum}\limits_{g\in P}\mathop{\sum}\limits_{s\in S}{M}_{0gs}.$$

**Fig. 6:**
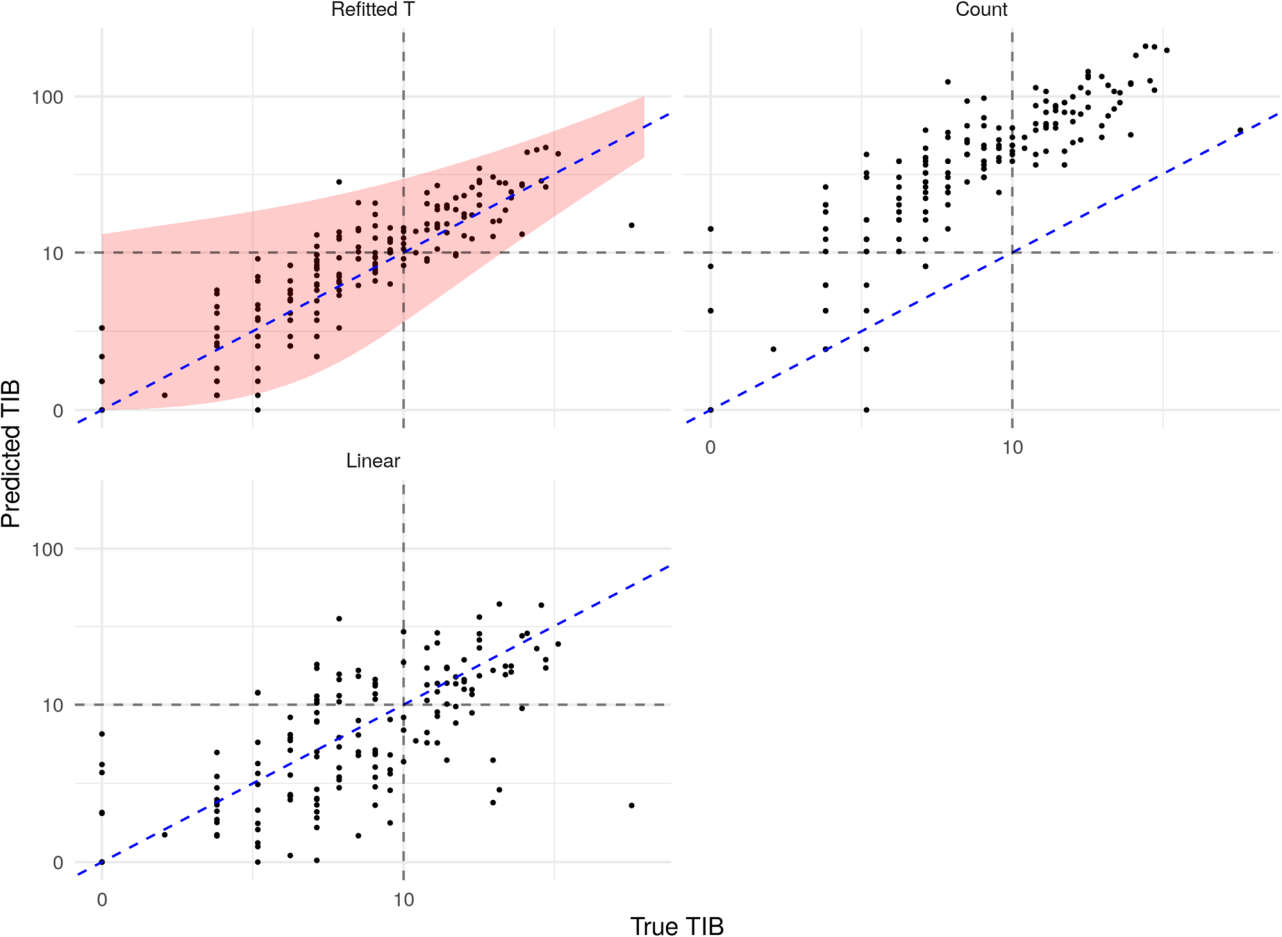
Estimation of TIB on the test dataset. Dashed blue (diagonal) line represents perfect prediction and the grey dashed lines indicate true and predicted TIB values of 10.

In Fig. 6 we present the predictions on the test set of our refitted estimator (*R*^2^ = 0.35); the count estimator (*R*^2^ = −44); and the linear regression estimator (*R*^2^ = −0.15). We also include (shaded in red) the set of points for which 90% prediction intervals contain the true value. In this case, we find that 97.7% of test set points fall within this region.

### External testing and classification of response to immunotherapy

The aim of this section is to further test our proposed estimator of TMB by making use of two external NSCLC datasets for which the response to immunotherapy is available: Hellmann et al.^21^, which contains 75 samples with an average TMB of 261; and Rizvi et al.^22^, which contains 34 samples with an average TMB of 258.

**Fig. 7:**
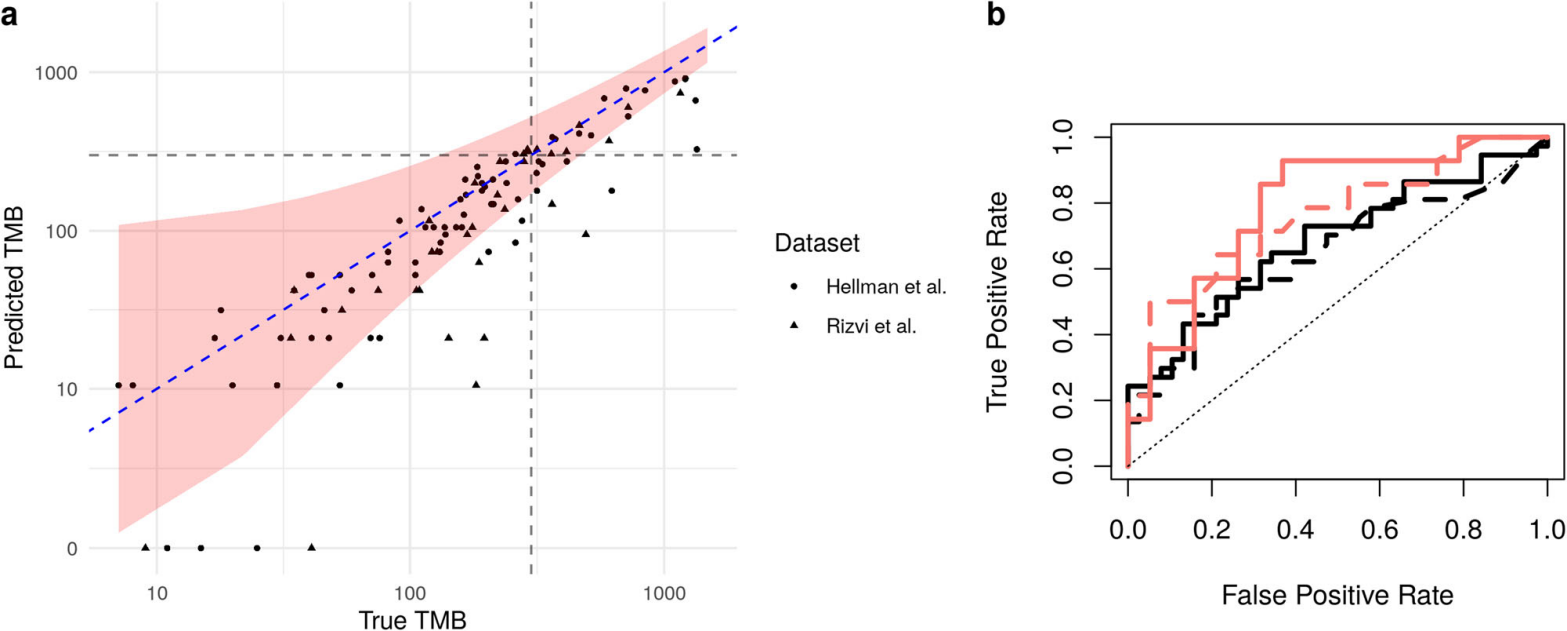
External test set regression and classification performance. **a** Performance of our model trained on the Campbell et al.^20^ dataset used to predict TMB based on the panel of size 0.6 Mb selected by our method on the external test datasets of Hellman et al.^21^ and Rizvi et al.^22^. **b** ROC curves for classifying the response to immunotherapy in the Hellman et al.^21^ (black) and Rizvi et al.^22^ (red) datasets using the true TMB values (solid) and estimated TMB values (dashed) based on the panel of size 0.6 Mb selected by our method.

We first use our refitted estimator trained on the same data as in the section above on predicting TMB to predict TMB for the samples in the new datasets using the selected panel of size 0.6 Mb. The predictions are given in Fig. 7a; the corresponding regression performance is *R*^2^ = 0.70 across the two datasets, with a joint AUPRC for classifying tumours to high or low TMB classes of 0.91.

These datasets also allow us to assess the practical utility of using our estimated TMB values to predict response to immunotherapy. Of the 75 samples in the Hellman et al.^21^ study, 37 were identified as having a *Durable Clinical Benefit* (Class 1) in response to immunotherapy (PD-L1+CTLA-4 blockade), and the remaining 38 were deemed to have *No Benefit* (Class 0). Of the 34 samples in the Rizvi et al.^22^ studies, 14 were identified as having a *Durable clinical benefit beyond 6 months* (Class 1) in response to immunotherapy (Pembrolizumab), while the remaining 20 were deemed not to have such benefit (Class 0). Since the treatment and outcome definitions differ between studies, we separate them for analysis of response. We construct two simple classifiers for comparison, the first assigning a sample to Class 1 if the true TMB value is greater than some threshold *t*, and the second using our estimated value of TMB in the same way. In Fig. 7b, we plot the receiver operating characteristic (ROC) curve (that is the false positive rate against the true positive rate as the classification threshold *t* varies). The area under the ROC curve is 0.68 for the Hellman et al.^21^ dataset when using the true TMB value and is 0.64 when using the estimated TMB value. Rizvi et al.^22^ have an area under the ROC curve of 0.79 using true TMB values and 0.76 using estimated TMB values. We see that, in both cases, very little is lost in terms of predicting response to immunotherapy when using our estimated value of TMB.

### Further testing in other cancer types

The aim of this section is to further demonstrate the performance of our proposed framework in a number of other cancer types. We apply our method for estimating TMB in six more cancer types, namely bladder cancer, breast cancer, colorectal cancer, melanoma, prostate cancer and renal cell cancer. For each cancer, data from two studies are used. Data from the first study is (randomly) split into a training and validation set; the training data is used to construct our estimator for a range of panel sizes, we then evaluate the predictive performance on the validation set (note that in contrast to our analysis above, we do not require a separate test set since the panel size is not selected based on the data). Further, in order to test the robustness of our approach to study effects, for each cancer type, we will also apply our fitted estimator (trained using data from the first study) to predict TMB values for tumours from the second study.

The twelve datasets^39–47^ used are detailed in Supplementary Table 2. These datasets have a range of mutation rates, specifically the average TMB values in the training datasets are 247 (bladder cancer), 91 (breast cancer), 339 (colorectal cancer), 568 (melanoma), 63 (prostate cancer) and 77 (renal cell cancer).

**Fig. 8:**
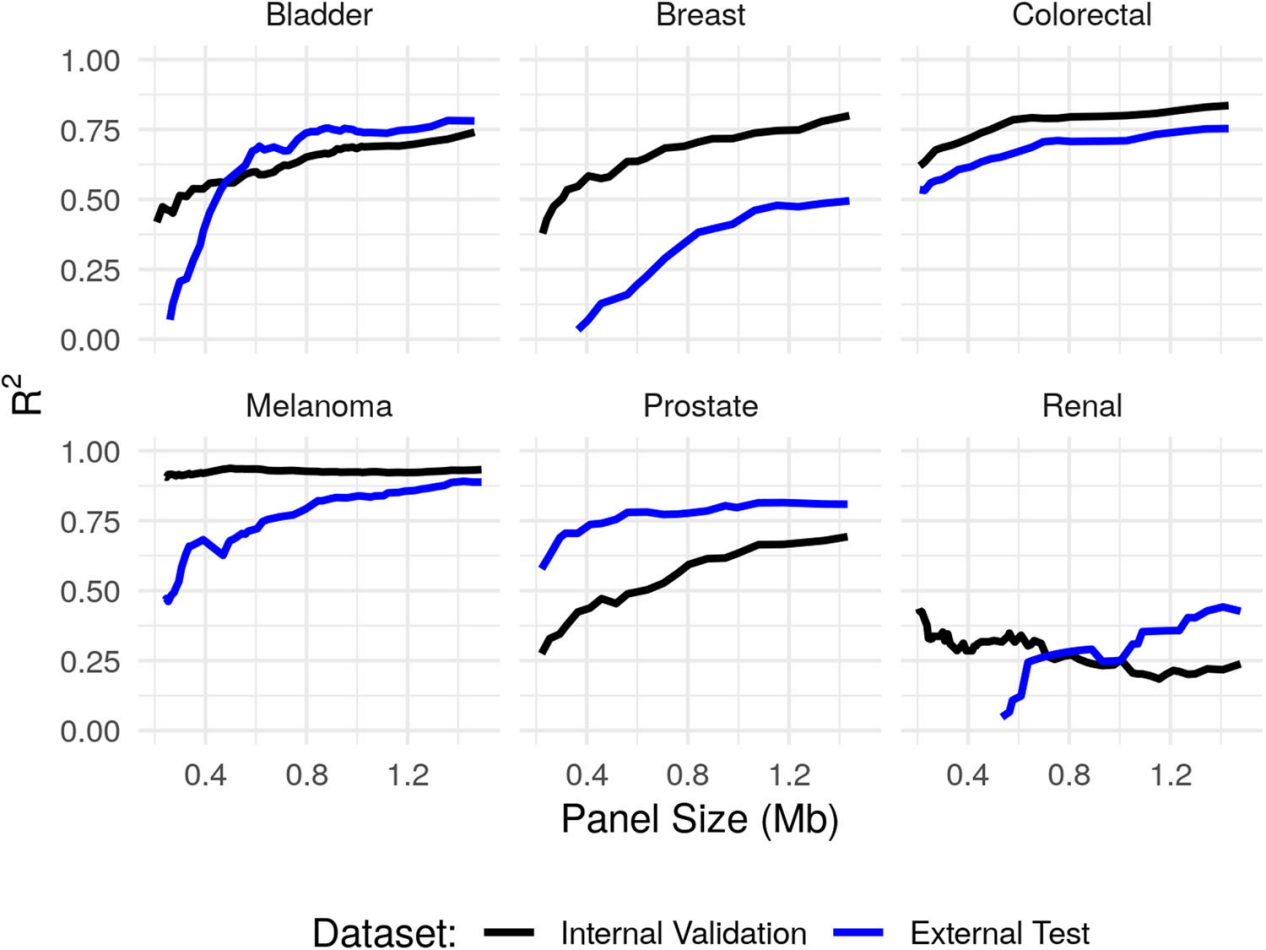
Predictive performance on six further cancer types. The performance of our refitted TMB estimator in the six further cancer types.

In Fig. 8, the black lines plot the *R*^2^ values obtained on the internal validation set from the first study for the six cancer types as the panel size varies from 0.25 Mb to 1.25 Mb. The blue lines show the *R*^2^ values obtained when predicting TMB for tumours in the external test set from the second study. We see that the performance on the internal validation set is very good and broadly in line with the performance we obtained for the NSCLC dataset (with the exception of renal cell cancer). The main factor affecting the performance appears to be the overall mutation rate; our method performs very well in cancer types with large mutation rates (colorectal cancer and melanoma), but less well in the cancers with lower overall mutation rates (prostate and renal cell). The performance on the renal cell dataset is particularly poor due to the combination of low sample size and the low average mutation rate.

The results on the external test datasets are more mixed; there is a drop-off in performance in comparison with the internal validation results for breast cancer and melanoma, but apparent improvement for prostate cancer. This highlights that study effects, such as differences in patient demographics and clinical profiles, as well as variations in sequencing technologies need to be considered carefully. In practice, one should ensure that the patients in the training data used to fit the model have similar characteristics to the intended test cohort.

## Conclusions

We have introduced a data-driven framework for designing targeted gene panels which allows for cost-effective estimation of exome-wide biomarkers. Using the non-small cell lung cancer datasets from Campbell et al.^20^, Hellman et al.^21^ and Rizvi et al.^22^, we have demonstrated the excellent predictive performance of our proposal for estimating tumour mutation burden and tumour indel burden, and shown that it outperforms the state-of-the-art procedures. We further tested the applicability and robustness of our method, by applying it to datasets on several other cancer types. Our framework can be applied to any tumour dataset containing annotated mutations, and we provide an R package^19^, which implements the methodology.

The main use of TMB is often to help identify patients that are more likely to respond to immunotherapy. While TMB is a good single predictor of response^10,11^, it is of course desirable to improve the predictive performance by including other factors. For instance, these may include cancer type (and subtype), specific mutational signatures, aneuploidy and tumor histology, as well as other variables, such as gender, age and exogenous factors. Indeed, Litchfield et al.^48^ show that, by including markers of T-cell infiltration and other factors, a multivariate predictor of response to immunotherapy significantly improves the classification performance in comparison to using TMB alone. Nevertheless, one would certainly like to include TMB (or a closely related measure) as a factor in any classifier of response.

Our work also has the scope to help understand mutational processes. For example, the parameters of our fitted model have interesting interpretations: of the five genes highlighted in Fig. 2a as having the highest mutation rates relative to the BMR, two (*TP53, CDKN2A*) are known tumour suppressors^49,50^ and *KRAS* is an oncogene^51^. Furthermore, indel mutations in *KRAS* are known to be deleterious for tumour cells^52^—in our work, the *KRAS* gene has a large negative indel-specific parameter (see Fig. 2b). Our methodology identifies a number of other genes with large parameter estimates. Of course, any such associations need to be carefully investigated in follow-up studies.

Finally, we believe there are many ways in which our general framework can be extended. For example, it may be adapted to incorporate alternate data types (e.g. transcriptomics); we may seek to predict other features (e.g. outcomes such as survival); or we may wish to extend the method to incorporate multiple potentially incomplete data sources.

## Methods

### Generative model

We now describe the main statistical model that underpins our methodology. In order to account for selective pressures and other factors within the tumour, we allow the rate at which mutations occur to depend on the gene and type of mutation. Our model also includes a sample-dependent parameter to account for the differing levels of mutagenic exposure of tumours, which may occur due to exogenous (e.g. UV light, cigarette smoke) or endogenous (e.g. inflammatory, free radical) factors.

We model the mutation counts *M*_*igs*_ as independent Poisson random variables with mutation rates *ϕ*_*igs*_ > 0. More precisely, for *i* = 0, 1, …, *n*, *g* ∈ *G* and *s* ∈ *S*, we have

2 $${M}_{igs} \sim {{{{{\rm{Poisson}}}}}}({\phi }_{igs}),$$

where *M*_*igs*_ and ${M}_{i^{\prime} g^{\prime} s^{\prime} }$ are independent for $(i,g,s)\,\ne\, (i^{\prime} ,g^{\prime} ,s^{\prime} )$. Further, to model the dependence of the mutation rate on the sample, gene and mutation type, we use a log link function and let

3 $$\log ({\phi }_{igs})={\mu }_{i}+\log ({\ell }_{g})+{\lambda }_{g}+{\nu }_{s}+{\eta }_{gs},$$

for ${\mu }_{i},{\lambda }_{g},{\nu }_{s},{\eta }_{gs}\in {\mathbb{R}}$, where for identifiability we set ${\eta }_{g{s}_{1}}=0$, for some *s*_1_ ∈ *S* and all *g* ∈ *G*.

The terms in our model can be interpreted as follows. First, the parameter *μ*_*i*_ corresponds to the BMR of the *i*th sample. The offset $\log ({\ell }_{g})$ accounts for a mutation rate that is proportional to the length of a gene, so that a non-zero value of *λ*_*g*_ corresponds to an increased or decreased mutation rate relative to the BMR. The parameters *ν*_*s*_ and *η*_*gs*_ account for differences in frequency between mutation types for each gene.

The model in (2) and (3) (discounting the unseen test sample *i* = 0) has *n* + ∣*S*∣ + ∣*G*∣∣*S*∣ free parameters and we have *n*∣*G*∣∣*S*∣ independent observations in the training dataset. In principle, we could attempt to fit our model directly using maximum likelihood estimation. However, we wish to exploit the fact that most genes do not play an active role in the development of a tumour, and will be mutated approximately according to the BMR. This corresponds to the parameters *λ*_*g*_ and *η*_*gs*_ being zero for many *g* ∈ *G*. We, therefore, include an *ℓ*_1_-penalisation term applied to the parameters *λ*_*g*_ and *η*_*gs*_ when fitting our model. We do not penalise the parameters *ν*_*s*_ or *μ*_*i*_ since we expect that different mutation types occur at different rates and that the BMR is different in each sample.

Writing *μ* ≔ (*μ*_1_, …, *μ*_*n*_), *λ* ≔ (*λ*_*g*_: *g* ∈ *G*), *ν* ≔ (*ν*_*s*_: *s* ∈ *S*) and *η* ≔ (*η*_*gs*_: *g* ∈ *G*, *s* ∈ *S*), and given training observations *M*_*igs*_ = *m*_*igs*_, we let

$${{{{{\mathcal{L}}}}}}(\mu ,\lambda ,\nu ,\eta )=\mathop{\sum }\limits_{i=1}^{n}\mathop{\sum}\limits_{g\in G}\mathop{\sum}\limits_{s\in S}\left({\phi }_{igs}-{m}_{igs}\log {\phi }_{igs}\right)$$

be the negative log-likelihood of the model specified by (2) and (3). We then define

4 $$(\hat{\mu },\hat{\lambda },\hat{\nu },\hat{\eta })=\mathop{{{\rm{argmin}}}}\limits_{\mu ,\lambda ,\nu ,\eta }\left\{{{{{{\mathcal{L}}}}}}(\mu ,\lambda ,\nu ,\eta )+{\kappa }_{1}\left(\mathop{\sum}\limits_{g\in G}| {\lambda }_{g}| +\mathop{\sum}\limits_{g\in G}\mathop{\sum}\limits_{s\in S}| {\eta }_{gs}| \right)\right\},$$

where *κ*_1_ ≥ 0 is a tuning parameter that controls the number of non-zero components in $\hat{\lambda }$ and $\hat{\eta }$, which we choose using cross-validation.

### Proposed estimator

We now attend to our main goal of estimating a given exome-wide biomarker for the unseen test sample. Fix $\bar{S}\subseteq S$ and recall that we write $T={T}_{0\bar{S}}$. We wish to construct an estimator of *T* that only depends on the mutation counts in a gene panel *P* ⊂ *G*, subject to a constraint on *ℓ*_*P*_. To that end, we consider estimators of the form

$$T(w):= \mathop{\sum}\limits_{g\in G}\mathop{\sum}\limits_{s\in S}{w}_{gs}{M}_{0gs},$$

for $w\in {{\mathbb{R}}}^{| G| \times | S| }$. Note that our estimator may use the full set *S* of variant types, rather than just those in $\bar{S}$. In other words, our estimator may utilise information from every mutation type, not just those that directly constitute the biomarker of interest. This is important when estimating mutation types in $\bar{S}$ that are relatively scarce (e.g. for TIB). In the remainder of this subsection, we explain how the weights *w* are chosen to minimise the expected squared error of *T*(*w*) based on the generative model described in the previous section.

Of course, setting *w*_*gs*_ = 1 for *g* ∈ *G* and $s\in \bar{S}$ (and *w*_*gs*_ = 0 otherwise) will give *T*(*w*) = *T*. However, our aim is to make predictions based on a concise gene panel. If, for a given *g* ∈ *G*, we have *w*_*gs*_ = 0 for all *s* ∈ *S*, then *T*(*w*) does not depend on the mutations in *g* and therefore the gene does not need to be included in the panel. In order to produce a suitable gene panel (i.e. with many *w*_*gs*_ = 0), we penalise non-zero components of *w* when minimising the expected squared error. We define our final estimator via a refitting procedure, which improves the predictive performance by reducing the bias, and is also helpful when applying our procedure to panels with predetermined genes.

To construct our estimator, note that under our model in (2) we have ${\mathbb{E}}{M}_{0gs}={{{{{\rm{Var}}}}}}({M}_{0gs})={\phi }_{0gs}$, and it follows that the expected squared error of *T*(*w*) is

5 $$\begin{array}{rcl}{\mathbb{E}}\left[{\{T(w)-T\}}^{2}\right]={{{{{\rm{Var}}}}}}(T(w))+{{{{{\rm{Var}}}}}}(T)-2{{{{{\rm{Cov}}}}}}(T(w),T)+{\left[{\mathbb{E}}\{T(w)-T\}\right]}^{2}\\ =\mathop{\sum}\limits_{g\in G}\mathop{\sum}\limits_{s\in \bar{S}}{(1-{w}_{gs})}^{2}{\phi }_{0gs}+\mathop{\sum}\limits_{g\in G}\mathop{\sum}\limits_{s\in S\setminus \bar{S}}{w}_{gs}^{2}{\phi }_{0gs}\\ +\,{\left(\mathop{\sum}\limits_{g\in G}\mathop{\sum}\limits_{s\in S}{w}_{gs}{\phi }_{0gs}-\mathop{\sum}\limits_{g\in G}\mathop{\sum}\limits_{s\in \bar{S}}{\phi }_{0gs}\right)}^{2}.\end{array}$$

This depends on the unknown parameters *μ*_0_, *λ*_*g*_, *ν*_*s*_ and *η*_*gs*_, the latter three of which are replaced by their estimates given in (4). It is also helpful to then rescale (5) as follows: write ${\hat{\phi }}_{0gs}={\ell }_{g}\exp ({\hat{\lambda }}_{g}+{\hat{\nu }}_{s}+{\hat{\eta }}_{gs})$, and define

$${p}_{gs}:= \frac{{\hat{\phi }}_{0gs}}{{\sum }_{g^{\prime} \in G}{\sum }_{s^{\prime} \in \bar{S}}{\hat{\phi }}_{0g^{\prime} s^{\prime} }}=\frac{{\ell }_{g}\exp ({\hat{\lambda }}_{g}+{\hat{\nu }}_{s}+{\hat{\eta }}_{gs})}{{\sum }_{g^{\prime} \in G}{\sum }_{s^{\prime} \in \bar{S}}{\ell }_{g^{\prime} }\exp ({\hat{\lambda }}_{g^{\prime} }+{\hat{\nu }}_{s^{\prime} }+{\hat{\eta }}_{g^{\prime} s^{\prime} })}.$$

Then let

$$f(w):= \mathop{\sum}\limits_{g\in G}\mathop{\sum}\limits_{s\in \bar{S}}{p}_{gs}{(1-{w}_{gs})}^{2}+\mathop{\sum}\limits_{g\in G}\mathop{\sum}\limits_{s\in S\setminus \bar{S}}{p}_{gs}{w}_{gs}^{2}+K({\mu }_{0}){\left(1-\mathop{\sum}\limits_{g\in G}\mathop{\sum}\limits_{s\in S}{p}_{gs}{w}_{gs}\right)}^{2},$$

where $K({\mu }_{0})=\exp ({\mu }_{0}){\sum }_{g\in G}{\sum }_{s\in \bar{S}}{\ell }_{g}\exp ({\hat{\lambda }}_{g}+{\hat{\nu }}_{s}+{\hat{\eta }}_{gs})$. Since *f* is a rescaled version of the error in (5) (with the true parameters *λ*, *ν*, *η* replaced by the estimates $\hat{\lambda },\hat{\nu },\hat{\eta }$), we will choose *w* to minimise *f*(*w*).

Note that *f* only depends on *μ*_0_ via the *K*(*μ*_0_) term, which can be interpreted as a penalty factor controlling the bias of our estimator. For example, we may insist that the squared bias term ${(1-{\sum }_{g\in G}{\sum }_{s\in S}{p}_{gs}{w}_{gs})}^{2}$ is zero by setting *K*(*μ*_0_) = *∞*. In practice, we propose to choose the penalty *K* based on the training data.

At this point *f*(*w*) is minimised by choosing *w* to be such that *w*_*gs*_ = 1 for all $g\in G,s\in \bar{S}$, and *w*_*gs*_ = 0 otherwise. As mentioned above, in order to form a concise panel while optimising predictive performance, we impose a constraint on the cost of sequencing the genes used in the estimation. More precisely, for a given *w*, an appropriate cost is

$$\parallel w{\parallel }_{G,0}:= \mathop{\sum}\limits_{g\in G}{\ell }_{g}{\mathbb{1}}\{{w}_{gs}\,\ne\, 0\,\,{{\mbox{for some}}}\,s\in S\}.$$

This choice acknowledges that the cost of a panel is roughly proportional to the length of the region of genomic space sequenced, and that once a gene has been sequenced for one mutation type there is no need to sequence again for other mutation types.

Now, given a cost restriction *L*, our goal is to minimise *f*(*w*) such that ∥*w*∥_*G*,0_ ≤ *L*. In practice, this problem is non-convex and so computationally infeasible. As is common in high-dimensional optimisation problems, we consider a convex relaxation as follows: let ∥*w*∥_*G*,1_ ≔ ∑_*g*∈*G*_*ℓ*_*g*_∥*w*_*g*_∥_2_, where ${w}_{g}=({w}_{gs}:s\in S)\in {{\mathbb{R}}}^{| S| }$, for *g* ∈ *G*, and ∥ ⋅ ∥_2_ is the Euclidean norm. Define

6 $${\hat{w}}^{{{{\mathrm{first-fit}}}}}\in \mathop{{{\rm{argmin}}}}\limits_{w}\left\{f(w)+{\kappa }_{2}\parallel w{\parallel }_{G,1}\right\},$$

where *κ*_2_ ≥ 0 is chosen to determine the size of the panel selected.

The final form of our estimator is obtained by a refitting procedure. First, for *P* ⊆ *G*, let

7 $${W}_{P}:= \{w\in {{\mathbb{R}}}^{| G| \times | S| }:{w}_{g}=(0,\ldots ,0)\,\,\,{{\mbox{for}}}\,\,\,g\in G\setminus P\}.$$

Let $\hat{P}:= \{g\in G:\,\parallel {\hat{w}}_{g}^{\,{{{{\rm{first-fit}}}}}\,}{\parallel }_{2} > 0\}$ be the panel selected by the first-fit estimator in (6), and define

8 $${\hat{w}}^{{{{{\mathrm{refit}}}}}}\in \mathop{{{\rm{argmin}}}}\limits_{w\in {W}_{\hat{P}}}\{f(w)\}.$$

We then estimate *T* using $\hat{T}:= T({\hat{w}}^{{{{{\rm{refit}}}}}})$, which only depends on mutations in genes contained in the selected panel $\hat{P}$.

### Panel augmentation

In practice, when designing gene panels a variety of factors contribute to the choice of genes included. For example, a gene may be included due to its relevance to immune response or its known association with a particular cancer type. If this is the case, measurements for these genes will be made regardless of their utility for predicting exome-wide biomarkers. When implementing our methodology, therefore, there is no additional cost to incorporate observations from these genes into our prediction if they will be helpful. Conversely, researchers may wish to exclude genes from a panel, or at least from actively contributing to the estimation of a biomarker, for instance, due to technical difficulties in sequencing a particular gene.

We can accommodate these restrictions by altering the structure of our regularisation penalty in (6). Suppose we are given (disjoint sets of genes) *P*_0_, *Q*_0_ ⊆ *G* to be included and excluded from our panel, respectively. In this case, we replace ${\hat{w}}^{{{{{\rm{first-fit}}}}}}$ in (6) with

9 $${\hat{w}}_{{P}_{0},{Q}_{0}}^{\,{{{{\mathrm{first-fit}}}}}\,}\in \mathop{{{\rm{argmin}}}}\limits_{w\in {W}_{G\setminus {Q}_{0}}}\left\{f(w)+{\kappa }_{2}\mathop{\sum}\limits_{g\in G\setminus {P}_{0}}{l}_{g}\parallel {w}_{g}{\parallel }_{2}\right\}.$$

Excluding the elements of *P*_0_ from the penalty term means that ${\hat{w}}_{{P}_{0},{Q}_{0}}^{\,{{{{\rm{first-fit}}}}}\,}\ne \,0$ for the genes in *P*_0_, while restricting our optimisation to ${W}_{G\setminus {Q}_{0}}$ excludes the genes in *Q*_0_ by definition. This has the effect of augmenting the predetermined panel *P*_0_ with additional genes selected to improve predictive performance. We then perform refitting as described above. We demonstrate this procedure by augmenting the TST-170 gene panel.

### Practical considerations

In this section, we discuss some practical aspects of our proposal. Our first consideration concerns the choice of the tuning parameter *κ*_1_ in (4). As is common for the Least Absolute Shrinkage and Selection Operator (LASSO) estimator in generalised linear regression (see, for example, Michoel^53^ and Friedman et al.^54^), we will use tenfold cross-validation. To highlight one important aspect of our cross-validation procedure, recall that we consider the observations *M*_*igs*_ as independent across the sample index *i* ∈ {1, …, *n*}, the gene *g* ∈ *G* and the mutation type *s* ∈ *S*. Our approach, therefore, involves splitting the entire set {(*i*, *g*, *s*): *i* = 1, …, *n*, *g* ∈ *G*, *s* ∈ *S*} of size *n*∣*G*∣∣*S*∣ (as opposed to the sample set {1, …, *n*}) into tenfolds uniformly at random. We then apply the estimation method in (4) to each of the tenfolds separately on a grid of values (on the log scale) of *κ*_1_, and select the value that results in the smallest average deviance across the folds. The model is then refitted using all the data for this value of *κ*_1_.

The estimated coefficients in (6) depend on the choice of *K*(*μ*_0_) and *κ*_2_. As mentioned above, we could set *K*(*μ*_0_) = *∞* to give an unbiased estimator, however, in practice, we found that a finite choice of *K*(*μ*_0_) leads to improved predictive performance. Our recommendation is to use $K({\mu }_{0})=K(\mathop{\max }\nolimits_{i=1,\ldots ,n}\{{\hat{\mu }}_{i}\})$, where ${\hat{\mu }}_{i}=\log ({T}_{i}/{\sum }_{g,s}{\ell }_{g}\exp ({\hat{\lambda }}_{g}+{\hat{\nu }}_{s}+{\hat{\eta }}_{gs}))$ is a pseudo-MLE (in the sense of Gong and Samaniego^55^) for *μ*_*i*_, so that the penalisation is broadly in proportion with the largest values of *μ*_*i*_ in the training dataset. The tuning parameter *κ*_2_ controls the size of the gene panel selected in (6): given a panel length *L*, we set ${\kappa }_{2}(L)=\max \{{\kappa }_{2}:\,{\ell }_{\hat{P}}\le L\}$ in order to produce a suitable panel.

We now comment briefly on some computational aspects of our method. The generative model fit in (4) can be solved via coordinate descent (see, for example, Friedman et al.^56^), which has a computational complexity of *O*(*N*∣*G*∣^2^∣*S*∣^2^) per iteration. We fit the model ten times, one for each fold in our cross-validation procedure. This is the most computationally demanding part of our proposal—in our experiments below, it takes approximately an hour to solve on a laptop—but it only needs to be carried out once for a given dataset. The convex optimisation problem in (6) can be solved by any method designed for the group LASSO; see, for example, Yang and Zou^57^. In our experiments, we use the gglasso R package^58^, which takes around 10 min to reproduce the plot in Supplementary Fig. 2. Note also that the solutions to (6) and (8) are unique; see, for example, Theorem 1 of Roth and Fischer^59^. The last step of our proposal, namely making predictions for new test observations based on a selected panel, carries a negligible computational cost.

Finally, we describe a heuristic procedure for producing prediction intervals around our point estimates. In particular, for a given confidence level *α* ∈ (0, 1), we aim to find an interval $[{\hat{T}}_{{{{{{\rm{L}}}}}}},{\hat{T}}_{{{{{{\rm{U}}}}}}}]$ such that ${\mathbb{P}}({\hat{T}}_{{{{{{\rm{L}}}}}}}\le T\le {\hat{T}}_{{{{{{\rm{U}}}}}}})\ge 1-\alpha .$ To that end, let ${t}_{\alpha }:= {\mathbb{E}}\{{(\hat{T}-T)}^{2}\}/\alpha$, then by Markov’s inequality we have that ${\mathbb{P}}(| \hat{T}-T{| }^{2}\ge {t}_{\alpha })\le \alpha$. It follows that $[\hat{T}-{t}_{\alpha }^{1/2},\hat{T}+{t}_{\alpha }^{1/2}]$ is a (1 − *α*)-prediction interval for *T*. Of course, the mean squared error ${\mathbb{E}}\{{(\hat{T}-T)}^{2}\}$ defined in (5) depends on the parameters *λ*, *η*, *ν* and *μ*_0_, which are unknown. Our approach is to utilise the estimates $\hat{\lambda },\hat{\eta },\hat{\nu }$ (see (4)) and replace *μ*_0_ with $\log (\hat{T}/{\sum }_{g,s}{\ell }_{g}\exp ({\hat{\lambda }}_{g}+{\hat{\nu }}_{s}+{\hat{\eta }}_{gs}))$. While this is not an exact (1 − *α*)-prediction interval for *T*, we see in our experimental results that in practice this approach provides intervals with valid empirical coverage.

### Reporting summary

Further information on research design is available in the Nature Research Reporting Summary linked to this article.

## Supplementary information

Peer Review File

Supplementary Material

Reporting Summary

## Data Availability

All data used in this manuscript are publicly available. The NSCLC dataset of Campbell et al.^20^ and the *Ensembl* gene length dataset are available as part of our R package ICBioMark^19^ - see below for more detail. The BED files for the gene panels can be downloaded from https://github.com/cobrbra/TargetedPanelEstimation_Paper^60^, while data citations for the six further cancer types are given in Supplementary Table 2.

## Code availability

All figures and tables in this manuscript may be reproduced using the code available at the DOI-minted repository https://github.com/cobrbra/TargetedPanelEstimation_Paper^60^. We also provide an open-access R package ICBioMark^19^, which is available on CRAN https://cran.r-project.org. Alternatively, the package may be accessed and downloaded at https://github.com/cobrbra/ICBioMark.
