# Supplementary material for: Data-driven design of targeted gene panels for estimating immunotherapy biomarkers
Source: Commun Biol. 2022 Feb 23;5:156. doi: 10.1038/s42003-022-03098-1 (PMC8866421; doi:10.1038/s42003-022-03098-1)
Supplement: Supplementary file 2 — Supplementary Material [file 42003_2022_3098_MOESM2_ESM.pdf]

Supplementary materials for 'Data-driven design of targeted gene panels for estimating immunotherapy biomarkers'

Jacob R. Bradley[1,*] and Timothy I. Cannings[1,†]
[1] *School of Mathematics, University of Edinburgh, Edinburgh, UK*
**j.r.j.bradley@sms.ed.ac.uk*, *†timothy.cannings@ed.ac.uk*

1 Generative model fitting

Supplementary Figure 1 shows the cross-validation computed deviance of our generative model as fitted to the NSCLC dataset[1] across a range of sparsity penalties.

Supplementary Figure 1: **Cross-validation of our generative model**

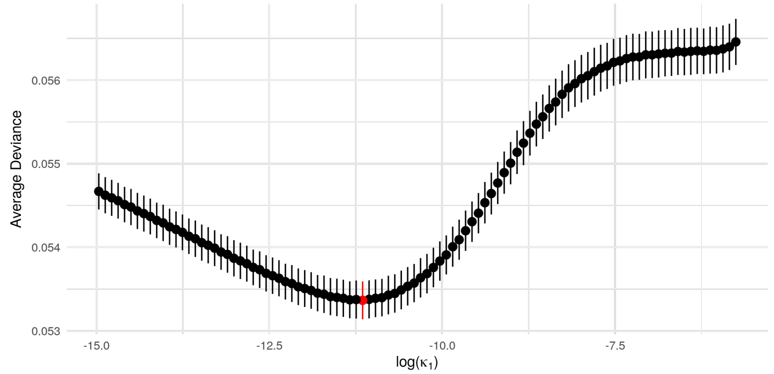

The average deviance (with one standard deviation) across the 10 folds in our cross-validation procedure plotted against $\log(\kappa_1)$. The minimum average deviance is highlighted red.

We validate our fitted model (as selected by taking the minimising penalty from Supplementary Figure 1) defined by equation (3) in the main text by comparing with the following alternatives:

(i) *Saturated model*: the model in equation (2), where each observation has an associated free parameter (i.e. $\phi_{igs} > 0$ is unrestricted);

(ii) *No sample-specific effects*: the model in equation (3), with $\mu_i = 0$ for all $i \in \{1, \dots, n\}$;

(iii) *No gene-specific effects*: the model in equation (3), with $\lambda_g = \eta_{gs} = 0$ for all $g \in G$ and $s \in S$;

(iv) *No gene/mutation type interactions*: the model in equation (3), with $\eta_{gs} = 0$ for all $g \in G$ and $s \in S$.

In Supplementary Table 1 we present the residual deviance and the residual degrees of freedom between our model and each of the models above. We see that our model is preferred over the saturated model, and all three submodels of (3).

Supplementary Table 1: Model comparisons on the basis of residual deviance statistics.

Comparison Model	Residual Deviance (dev)	Residual Degrees of Freedom (df)	dev/df	p-value
(i)	1.43×10^6	2.74×10^7	5.22×10^{-2}	1.00
(ii)	1.42×10^5	8.00×10^2	1.77×10^2	0.00
(iii)	1.10×10^5	1.33×10^4	8.24×10^0	0.00
(iv)	1.70×10^4	1.82×10^3	9.33×10^0	0.00

2 Full validation set performance for prediction TMB

Supplementary Figure 2: **Validation set performance of estimators**

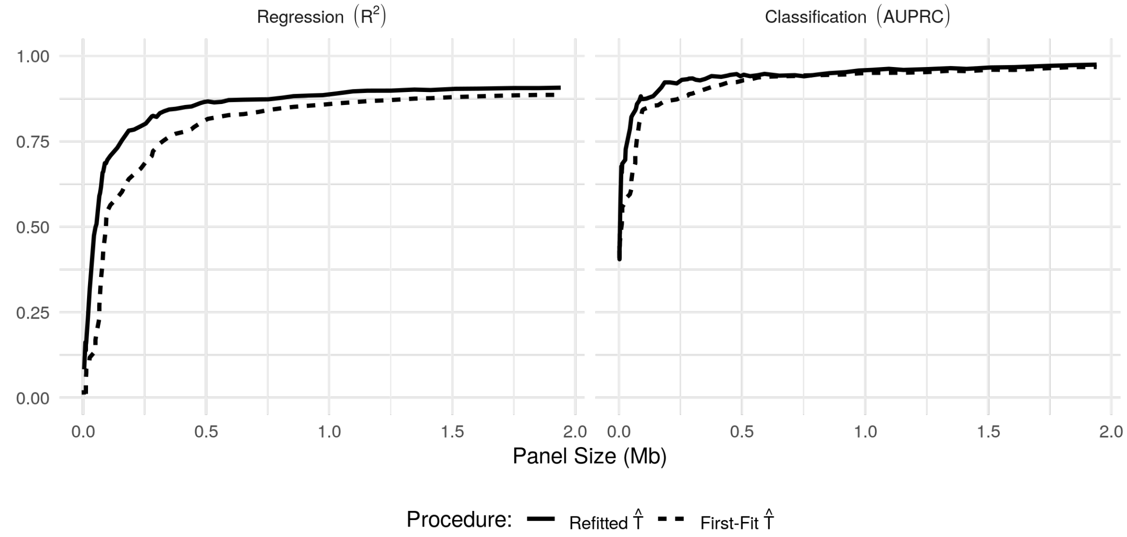

Performance of our first-fit and refitted estimators of Tumour Mutation Burden (TMB) as the selected panel size varies. **Left**: R^2, **Right**: area under the precision-recall curve (AUPRC).

3 Predictive model robustness

Here we investigate the robustness of our proposal to changes in the training dataset. We conduct an experiment that first involves splitting the training data set of $n = 800$ observations into four disjoint datasets of 200 observations. We then retrain our model and estimator (described in Section 2 of the main text) based on the four possible datasets that combine three of the four subsets. We then evaluate the predictive performance on the validation dataset similarly to the analysis shown in Figure 3 of the main text. The results are given in

Supplementary Figure 3; we see that our proposal is has very similar regression performance on the four different subsets.

Supplementary Figure 3: **Robustness to the training dataset**

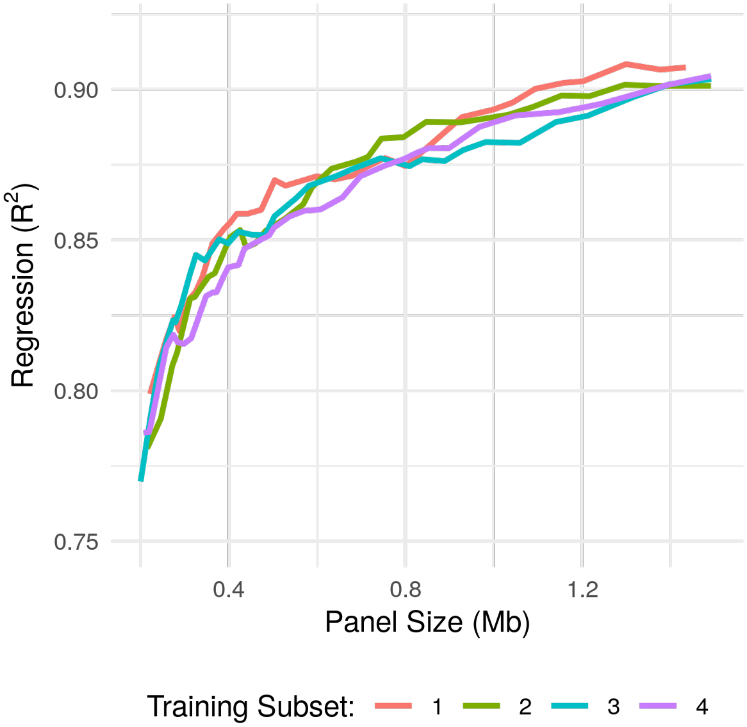

The regression performance on the validation dataset for the four different training subsets of 600 observations.

4 External data sources

Supplementary Table 2: Description of external datasets used for multi-cancer comparisons.

Cancer Type	Author(s)	Year	n_{train}	n_{val}	n_{test}
Bladder	TCGA Pan-Cancer Atlas[1]	2018	300	109	0
Bladder	Guo et al.[2]	2013	0	0	99
Breast	TCGA Pan-Cancer Atlas[2]	2018	700	300	0
Breast	Kan et al.[3]	2018	0	0	187
Colorectal	Giannakis et al.[4]	2016	500	119	0
Colorectal	Seshagiri et al.[5]	2012	0	0	72
Melanoma	The Cancer Genome Atlas Network[6]	2015	250	96	0
Melanoma	Krauthammer et al.[7]	2012	0	0	91
Prostate	Armenia et al.[8]	2018	700	312	0
Prostate	Kumar et al.[9]	2016	0	0	141
Renal	TCGA Firehose[3]	-	350	101	0
Renal	Guo et al.[10]	2011	0	0	98

[1] data available at https://www.cbioportal.org/study/clinicalData?id=blca_tcga_pan_can_atlas_2018

[2] data available at https://www.cbioportal.org/study/summary?id=brca_tcga_pan_can_atlas_2018

[3] data available at https://www.cbioportal.org/study/summary?id=kirc_tcga

References

[1] Campbell, J. D. *et al.* Distinct patterns of somatic genome alterations in lung adenocarcinomas and squamous cell carcinomas. *Nature Genetics* **48**, 607–616 (2016).

[2] Guo, G. *et al.* Whole-genome and whole-exome sequencing of bladder cancer identifies frequent alterations in genes involved in sister chromatid cohesion and segregation. *Nature Genetics* **45**, 1459–1463 (2013).

[3] Kan, Z. *et al.* Multi-omics profiling of younger Asian breast cancers reveals distinctive molecular signatures. *Nature Communications* **9**, 1725 (2018).

[4] Giannakis, M. *et al.* Genomic Correlates of Immune-Cell Infiltrates in Colorectal Carcinoma. *Cell Reports* **15**, 857–865 (2016).

[5] Seshagiri, S. *et al.* Recurrent R-spondin fusions in colon cancer. *Nature* **488**, 660–664 (2012).

[6] Cancer Genome Atlas Network. Genomic Classification of Cutaneous Melanoma. *Cell* **161**, 1681–1696 (2015).

[7] Krauthammer, M. *et al.* Exome sequencing identifies recurrent somatic RAC1 mutations in melanoma. *Nature Genetics* **44**, 1006–1014 (2012).

[8] Armenia, J. *et al.* The long tail of oncogenic drivers in prostate cancer. *Nature Genetics* **50**, 645–651 (2018).

[9] Kumar, A. *et al.* Substantial interindividual and limited intraindividual genomic diversity among tumors from men with metastatic prostate cancer. *Nature Medicine* **22**, 369–378 (2016).

[10] Guo, G. *et al.* Frequent mutations of genes encoding ubiquitin-mediated proteolysis pathway components in clear cell renal cell carcinoma. *Nature Genetics* **44**, 17–19 (2011).
