# Supplementary material for: Data-driven design of targeted gene panels for estimating immunotherapy biomarkers
Source: Commun Biol. 2022 Feb 23;5:156. doi: 10.1038/s42003-022-03098-1 (PMC8866421; doi:10.1038/s42003-022-03098-1)
Supplement: Supplementary file 3 — Reporting Summary [file 42003_2022_3098_MOESM3_ESM.pdf]

nature portfolio

Corresponding author(s): Timothy I. Cannings

Last updated by author(s): Dec 21, 2021

Reporting Summary

Nature Portfolio wishes to improve the reproducibility of the work that we publish. This form provides structure for consistency and transparency in reporting. For further information on Nature Portfolio policies, see our Editorial Policies and the Editorial Policy Checklist.

Statistics

For all statistical analyses, confirm that the following items are present in the figure legend, table legend, main text, or Methods section.

n/a	Confirmed	
☐	☒	The exact sample size (*n*) for each experimental group/condition, given as a discrete number and unit of measurement
☐	☒	A statement on whether measurements were taken from distinct samples or whether the same sample was measured repeatedly
☐	☒	The statistical test(s) used AND whether they are one- or two-sided<br>*Only common tests should be described solely by name; describe more complex techniques in the Methods section.*
☐	☒	A description of all covariates tested
☐	☒	A description of any assumptions or corrections, such as tests of normality and adjustment for multiple comparisons
☐	☒	A full description of the statistical parameters including central tendency (e.g. means) or other basic estimates (e.g. regression coefficient) AND variation (e.g. standard deviation) or associated estimates of uncertainty (e.g. confidence intervals)
☐	☒	For null hypothesis testing, the test statistic (e.g. *F*, *t*, *r*) with confidence intervals, effect sizes, degrees of freedom and *P* value noted<br>*Give P values as exact values whenever suitable.*
☒	☐	For Bayesian analysis, information on the choice of priors and Markov chain Monte Carlo settings
☒	☐	For hierarchical and complex designs, identification of the appropriate level for tests and full reporting of outcomes
☒	☐	Estimates of effect sizes (e.g. Cohen's *d*, Pearson's *r*), indicating how they were calculated

Our web collection on statistics for biologists contains articles on many of the points above.

Software and code

Policy information about availability of computer code

Data collection	All data used in this manuscript is publicly available; and code used to collect data is available in the R package ICBioMark (https://github.com/cobrbra/ICBioMark).
Data analysis	All figures and tables in the manuscript may be reproduced using the code available at https://github.com/cobrbra/TargetedPanelEstimation_Paper. We also provide an open access R package ICBioMark, which is available on CRAN (https://cran.r-project.org). Alternatively, the package may be accessed and downloaded at https://github.com/cobrbra/ICBioMark.

For manuscripts utilizing custom algorithms or software that are central to the research but not yet described in published literature, software must be made available to editors and reviewers. We strongly encourage code deposition in a community repository (e.g. GitHub). See the Nature Portfolio guidelines for submitting code & software for further information.

Data

Policy information about availability of data

All manuscripts must include a data availability statement. This statement should provide the following information, where applicable:

- Accession codes, unique identifiers, or web links for publicly available datasets
- A description of any restrictions on data availability
- For clinical datasets or third party data, please ensure that the statement adheres to our policy

All data used in this manuscript is publicly available. The NSCLC dataset of Campbell et al. (2016) and the Ensembl gene length dataset are available as part of our R package ICBioMark. The BED files for the gene panels used in Section 3.2 of the paper can be downloaded from https://github.com/cobrbra/TargetedPanelEstimation_Paper.

March 2021

Field-specific reporting

Please select the one below that is the best fit for your research. If you are not sure, read the appropriate sections before making your selection.

☒ Life sciences ☐ Behavioural & social sciences ☐ Ecological, evolutionary & environmental sciences

For a reference copy of the document with all sections, see nature.com/documents/nr-reporting-summary-flat.pdf

Life sciences study design

All studies must disclose on these points even when the disclosure is negative.

Sample size	The sample sizes were determined by the size of data sets available.
Data exclusions	No data were excluded.
Replication	All figures and tables in the manuscript may be reproduced using the code available at https://github.com/cobrbra/TargetedPanelEstimation_Paper.
Randomization	All allocations were made at random.
Blinding	Blinding was not relevant since all data is publicly available -- all appropriate measures (i.e. separation of the data into training, validation and test sets) are described in detail the manuscript.

Reporting for specific materials, systems and methods

We require information from authors about some types of materials, experimental systems and methods used in many studies. Here, indicate whether each material, system or method listed is relevant to your study. If you are not sure if a list item applies to your research, read the appropriate section before selecting a response.

Materials & experimental systems

n/a	Involved in the study	
☒	☐	Antibodies
☒	☐	Eukaryotic cell lines
☒	☐	Palaeontology and archaeology
☒	☐	Animals and other organisms
☒	☐	Human research participants
☒	☐	Clinical data
☒	☐	Dual use research of concern

Methods

n/a	Involved in the study	
☒	☐	ChIP-seq
☒	☐	Flow cytometry
☒	☐	MRI-based neuroimaging
